# Streptococcus pyogenes Infection-Induced Primary Peritonitis in a Healthy Adult Female: A Very Rare Causative Agent

**DOI:** 10.7759/cureus.43330

**Published:** 2023-08-11

**Authors:** Koichi Soga, Mika Mazaki, Shun Takakura, Hiroaki Kitae, Naoaki Akamatsu

**Affiliations:** 1 Department of Gastroenterology, Dokkyo Medical University Saitama Medical Center, Saitama, JPN; 2 Department of Gastroenterology, Omihachiman Community Medical Center, Shiga, JPN

**Keywords:** streptococcus pyogenes, primary peritonitis, peritonitis, group a streptococcus, acute abdomen

## Abstract

A 44-year-old woman with an unremarkable medical history presented to another hospital complaining of lower abdominal pain and nausea. The clinical presentation was consistent with an acute abdomen, raising suspicion of gastrointestinal tract perforation. However, imaging studies failed to provide evidence of perforation. Subsequently, the patient was diagnosed with peritonitis of unknown origin and promptly administered broad-spectrum antibiotics in a fasting state. Although the patient initially exhibited unstable symptoms, hemodynamics, and serology, she gradually improved over three days, with values approaching normal levels. On the sixth day of hospitalization, a follow-up abdominal computed tomography scan revealed pleural effusions, extensive ascites, and intra-abdominal stranding. The thickened wall of the small intestine and intra-abdominal stranding that were suggestive of peritonitis were further exacerbated. On the seventh day of hospitalization, aerobic and anaerobic blood cultures revealed the presence of Gram-positive cocci, later confirmed to be *Streptococcus pyogenes*, leading to the diagnosis of *S. pyogenes* infection-induced primary peritonitis. The source of infection was identified as a 10 mm hydrosalpinx in the left fallopian tube, suggesting the possibility of retrograde infection. The patient ultimately made a complete recovery without relapse and has been doing well since. This case report highlights a unique and rare occurrence of primary peritonitis caused by group A Streptococcus associated with infection from a hydrosalpinx in an otherwise healthy and young female patient. The diagnosis of primary spontaneous bacterial peritonitis in such an individual presents an uncommon clinical manifestation, emphasizing the importance of considering atypical sources of peritoneal infection in clinical practice.

## Introduction

Acute abdominal pain in individuals with no prior medical history is a rare presentation of primary spontaneous bacterial peritonitis. Primary peritonitis, defined as peritonitis occurring without an intra-abdominal source, is an infrequent condition. Group A Streptococcus (GAS), also called *Streptococcus pyogenes*, usually causes pharyngitis, erysipelas, and necrotizing fasciitis. However, reports show that GAS infections are becoming more common and more severe [1]. However, *S. pyogenes*-related primary peritonitis is uncommon and rarely diagnosed in healthy individuals without underlying diseases. Hydrosalpinx, characterized by a collection of fluid in the fallopian tube lumen, usually manifests without symptoms. It can be caused by sexually transmitted infections, endometriosis, previous fallopian tube surgery, or a fallopian tube infection [2]. In this report, we present a rare case of unusual acute primary peritonitis due to GAS in a 44-year-old healthy patient. Blood cultures revealed the presence of Gram-positive cocci, later confirmed to be *S. pyogenes*.

## Case presentation

A 44-year-old woman with no prior medical history was admitted to the general ward of a different hospital with complaints of lower abdominal pain and nausea. The patient underwent fluid rehydration therapy (1500 mL/day of a hypotonic electrolyte solution) and received intravenous administration of meropenem (0.5 g thrice daily) for suspected severe gastroenteritis. Despite these interventions, her condition continued to worsen, necessitating her transfer to our hospital. Upon admission, she developed diffuse abdominal pain with peritoneal irritation, predominantly localized in the epigastric fossa. The physical examination did not reveal any other apparent sources of infection. The patient had not taken any medication, traveled, or consumed perishable food, nor did she report any respiratory or urinary symptoms.

**Figure 1:**
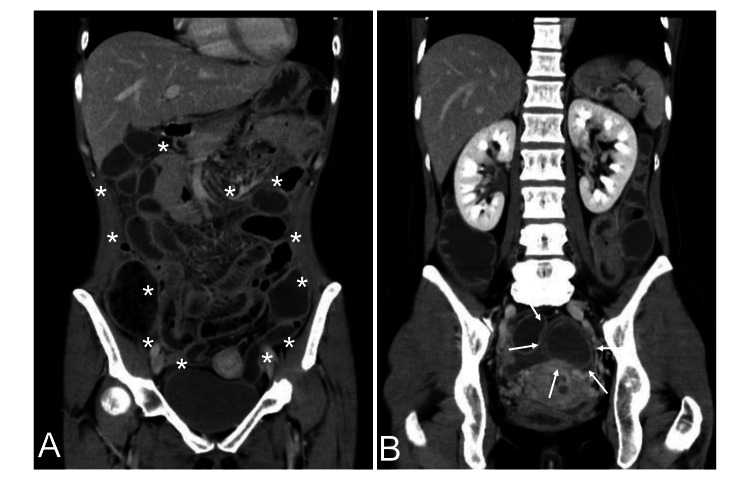
Previously healthy 44-year-old woman entered the general ward of another hospital complaining of lower abdominal pain and nausea. Abdominal computed tomography showing a thickened wall of the entire small intestine (A; asterisk) and ascites in the Douglas fossa (B; arrow).

Upon admission to our hospital, the patient exhibited vital signs indicative of an altered physiological state, with a heart rate of 103 beats/min, blood pressure of 92/50 mmHg, oxygen saturation of 99% while breathing ambient air, and a temperature of 38.4 °C. Blood tests demonstrated the following findings: leukocyte count of 36,400 /μL, hemoglobin level of 11.0 g/dL, platelet count of 324,000 /μL, C-reactive protein level of 21.86 mg/dL, creatinine level of 0.85 mg/dL, and procalcitonin level of 11.83 ng/mL. Blood gas analysis revealed an elevated arterial lactate level of 28.0 mg/dL. Her antinuclear antibody was noted to be 160-fold, while the serum antibodies against Chlamydia trachomatis were >10 cut off index (COI) for IgG and 0.71 COI within the reference range for IgA. Polymerase chain reaction tests for chlamydia and gonorrhea performed on vaginal mucosal specimens yielded negative results. Additionally, both influenza and COVID-19 antigen tests conducted using pharyngeal mucosal specimens were negative. Abdominal computed tomography (CT) scans revealed thickening of the entire small intestine wall (Figure 1A), ascites in the Douglas fossa (Figure 1B), and intra-abdominal stranding, indicative of peritonitis.

The patient's presentation was deemed indicative of an acute abdomen with suspected perforation of the gastrointestinal tract. However, imaging studies failed to reveal any findings suggestive of perforation or free air. Following extensive deliberation amongst our hospital staff, the patient was diagnosed with peritonitis of unknown etiology. Treatment was initiated with strict control, consisting of broad-spectrum antibiotics (meropenem, 0.5 g every eight hours) and fasting. Initially, the patient exhibited unstable symptoms, hemodynamics, and serological parameters; however, improvement was observed over three days with a gradual return to normal values.

**Figure 2:**
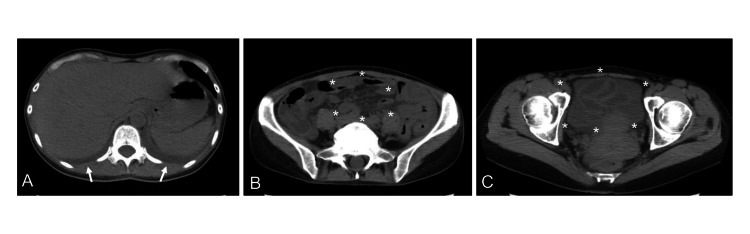
A follow-up abdominal CT scan conducted six days after admission to our hospital. Follow-up abdominal CT scan performed six days after hospital admission, showing pleural effusions (A; arrow), intra-abdominal stranding (B; asterisk), and extensive ascites (C; asterisk).

**Figure 3:**
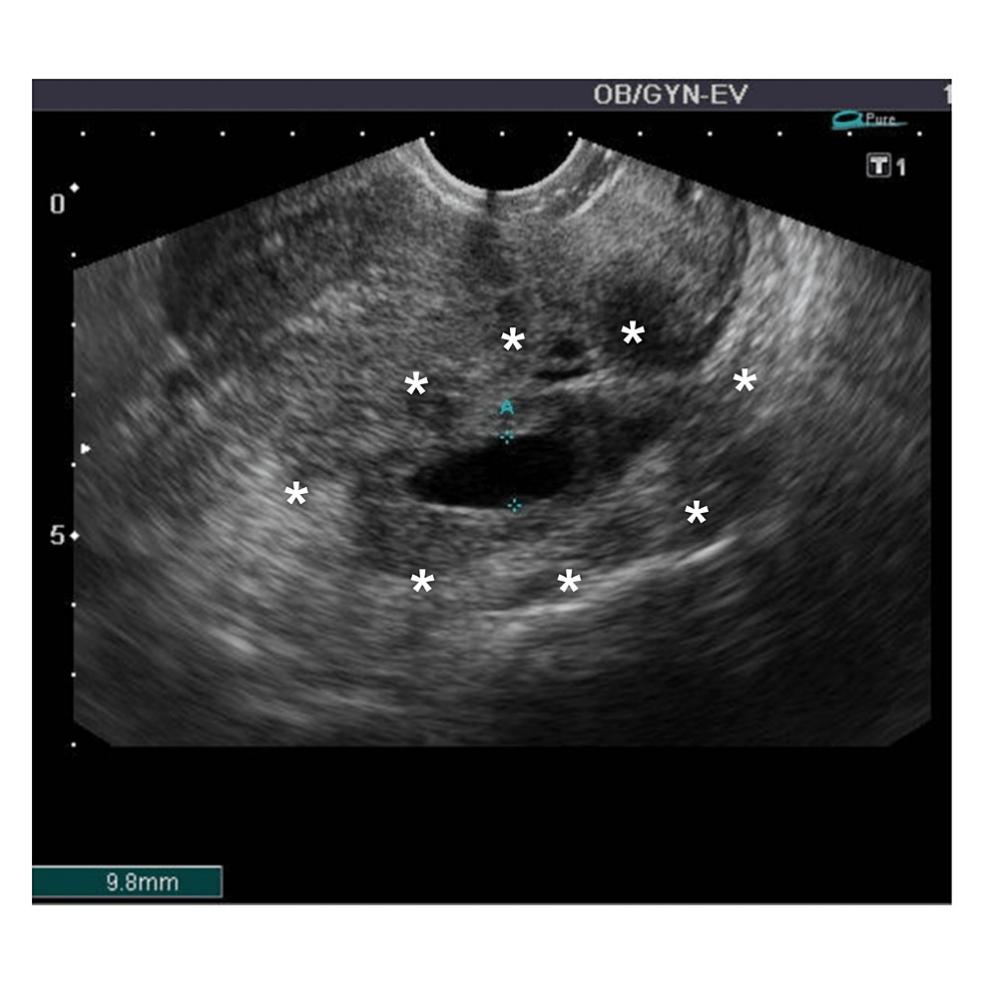
Hydrosalpinx of 9.8 mm in the left fallopian tube. Hydrosalpinx of 9.8 mm in the left fallopian tube (asterisk), suggesting the possibility of retrograde infection, found by a gynecologist while identifying the source of group A streptococcus infection.

A follow-up abdominal CT scan conducted six days after admission to our hospital revealed the presence of pleural effusions (Figure 2A), intra-abdominal stranding (Figure 2B), and extensive ascites (Figure 2C). These findings were consistent with the exacerbation of the thickened small intestinal wall and peritonitis. However, no other intra-abdominal pathology or free air was observed on the scan. Seven days following hospital admission, Gram-positive cocci were identified in blood cultures conducted under both aerobic and anaerobic conditions. Subsequent culture analysis confirmed the presence of GAS. The patient's treatment was altered to sulbactam/ampicillin (3.0 g every eight hours) following confirmation of the GAS infection. In an effort to ascertain the origin of the infection, a gynecologist was consulted, and a 10 mm hydrosalpinx was identified in the left fallopian tube, suggesting the possibility of retrograde infection (Figure 3). Therefore, it was determined that the peritonitis was caused by GAS infected with hydrosalpinx.

**Figure 4:**
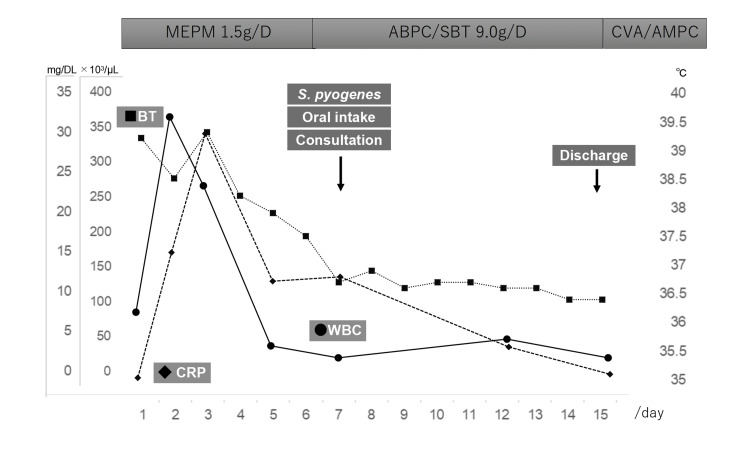
The clinical course of the patient. BT: body temperature; CRP: C-reactive protein; MEPM: meropenem; SBT/ABPC: sulbactam/ampicillin.

After completing the intravenous antibiotic regimen, the patient was transitioned to oral antibiotics consisting of amoxicillin and clavulanic acid with supplemental amoxicillin (250 mg after every meal) and discharged with a prescription to complete the oral antibiotics for one week. The total duration of antibiotic therapy was 22 days, comprising 7 days of meropenem, 8 days of sulbactam/ampicillin, and 7 days of oral amoxicillin and clavulanic acid with supplemental amoxicillin. Subsequently, the patient made a complete recovery and did not experience any relapses. After her symptoms had improved, the gynecologist was consulted, and treatment strategies, including salpingectomy, were considered. The clinical course of the patient is represented in Figure 4.

## Discussion

This is a unique instance of primary peritonitis, commonly referred to as spontaneous bacterial peritonitis, characterized by peritoneal infection without a discernible intra-abdominal origin. The etiology of primary peritonitis is postulated to originate from hematogenous, lymphatic, bowel translocation, or genital tract sources [3].

Peritonitis can be classified into three main types: primary, secondary, and tertiary, with secondary being the more common of the three [4]. Primary peritonitis accounts for only 1% of all peritonitis cases, making it a very rare disease among otherwise healthy individuals without comorbidities. However, it is usually associated with patients having underlying autoimmune diseases such as systemic lupus erythematosus, immunosuppression, chronic liver disease with ascites, or chronic kidney disease [5]. Conversely, secondary peritonitis frequently results from intra-abdominal lesions such as bowel perforation and ischemia. Lastly, tertiary peritonitis is characterized by persistent or recurrent infection after 48 hours following successful and adequate surgical source control. It typically involves organisms of low intrinsic virulence and affects immunocompromised patients, leading to progressive organ dysfunction and, consequently, high mortality [6].

The initial clinical presentation of primary GAS peritonitis is multifaceted and can range from isolated peritonitis without hemodynamic compromise or indications of streptococcal infection to severe sepsis, necrotizing fasciitis, and streptococcal toxic shock syndrome (STSS) [7]. The pathophysiological underpinnings of this variant of peritonitis remain incompletely elucidated. According to a systematic review of GAS primary peritonitis, 15.6% of patients manifested an ascending infection originating from the vaginal region, 15.6% of patients exhibited droplet transmission as the source of infection, and 59.3% of cases had an unknown route of transmission. Furthermore, 6.3% of cases were associated with fasciitis, while 3.1% of cases had insect bites as the source of infection [8]. Iitaka et al. documented 46 cases of GAS primary peritonitis in adults, of which 87.0% (40 cases) pertained to females, with a median age of 40.0 years and an age range of 23-87 years [9]. Notably, the higher incidence of GAS primary peritonitis among women may potentially be attributed to ascending infections from the genitourinary tract [7].

In the present case, the initial diagnosis of gastroenteritis should have been considered a precipitating factor in the patient's presentation, even though gastroenteritis caused by GAS infection is a rare occurrence. The possibility of gastroenteritis stemming from swallowing GAS from the upper respiratory tract cannot be entirely ruled out, considering the gastrointestinal symptoms of nausea and vomiting. As the patient did not have diarrhea symptoms, gastroenteritis was considered negative. However, based on the imaging findings, we posit that the hydrosalpinx became infected, resulting in a pelvic infection, and the inflammation subsequently disseminated to the retroperitoneal duodenum via the retroperitoneum, leading to upper abdominal symptoms of nausea and vomiting. While there is no definitive confirmation of a gastrointestinal tract infection, the intestinal migration of GAS from a focal respiratory tract infection may indirectly cause gastroenteritis [10].

Hydrosalpinx can develop after the onset of post-surgical adhesions or after a hysterectomy, but more commonly, it is related to a previous episode of acute pelvic inflammatory disease (PID) [11]. Fallopian tube infection leads to inflammation and PID, increasing a woman's risk for ectopic pregnancy by 9%, tubal-factor infertility by 16% [12], and causing chronic pelvic pain in 36% of patients [13]. In a study, Taipale et al. monitored patients diagnosed with acute PID for three months, and after this period, 26.7% of them showed signs of hydrosalpinx during sonographic examination [14].

Primary GAS peritonitis poses diagnostic challenges and is typically diagnosed through a process of exclusion. The mainstay of managing peritonitis involves prompt diagnosis, early empirical antibiotic treatment, and the careful exclusion of secondary peritonitis, which may require more aggressive and early surgical intervention. Imaging techniques such as ultrasound and CT scans play a crucial role in identifying possible underlying intra-abdominal pathology, reducing the need for immediate surgical exploration. Additionally, a thorough physical examination is essential to identifying potential primary sources of peritonitis [4].

Regarding the clinical presentation, a majority of reported cases in the literature present with severe abdominal pain and a high fever, prompting suspicion of secondary peritonitis [15]. However, it was arduous to determine the portal of entry based on symptoms or a physical examination in this case. The usefulness of surgical exploration in patients with negative CT scans, where secondary peritonitis cannot be definitively ruled out, remains unclear [16]. Treatment of GAS peritonitis frequently involves surgical intervention, with 88.4% of patients undergoing surgery, primarily through laparotomy (65.1%) or laparoscopy (23.3%), while only 11.6% of patients receive antibiotic therapy alone [9].

This case was deemed an acute abdomen; nonetheless, given the absence of definitive findings to warrant surgical intervention, the patient was managed conservatively while being closely monitored. The diagnosis of primary peritonitis is commonly established retrospectively, following the exclusion of other causes via surgery, as abdominal CT scans typically reveal only intraperitoneal fluid, which is a non-specific finding in most cases. Abdominal CT reliably eliminates secondary peritonitis from an intra-abdominal source in patients with acute abdominal pain of unknown etiology [17]. Additionally, surgical exploration aids in ruling out differential diagnoses such as perforated diverticula or appendicitis [8]. Consequently, most patients are subjected to exploratory laparotomy or laparoscopy with a preoperative diagnosis of gastrointestinal perforation. In this particular case, upon confirming GAS from the blood culture, immediate consultation with the gynecologist and subsequent transvaginal ultrasonography revealed hydrosalpinx as the source of infection.

Antimicrobial therapy should be initiated promptly [18]. Nonetheless, the literature provides limited and inconsistent information regarding the optimal antibiotic regimens for treating GAS peritonitis [19]. For uncomplicated GAS infections, penicillin is the preferred option due to its well-established efficacy against beta-lactam-sensitive GAS strains [20]. In contrast, broad-spectrum antimicrobials are recommended for septic patients. The necessity for surgical intervention must be cautiously evaluated based on the severity of peritonitis following the prompt institution of appropriate antimicrobial therapy.

Our case highlights the occurrence of primary SBP in a healthy and young woman. Primary SBP is less common than SBP overall and can be challenging to diagnose and manage effectively. It is crucial for gastroenterologists, general practitioners, and surgeons to be aware of cases involving primary SBP due to GAS and associated with infection from hydrosalpinx.

## Conclusions

*Streptococcus pyogenes* (GAS) infection is a relatively uncommon cause of primary peritonitis, with a higher prevalence observed among middle-aged women. In such cases, maintaining a high clinical suspicion and ensuring prompt diagnosis, alongside meticulous monitoring, constitute essential aspects of patient care when encountering symptoms and signs of acute abdomen. The cornerstone of treatment, involving the timely administration of empirical antibiotics, must be promptly initiated, and the source of infection should be methodically investigated. In this particular case, surgical intervention was deemed unnecessary due to the lack of compelling evidence, thereby avoiding an unwarranted operation. A strict follow-up protocol and comprehensive evaluation of primary peritonitis were implemented to ensure optimal patient management. It is worth noting that the majority of patients with hydrosalpinx remain asymptomatic. Furthermore, in cases of peritonitis with an unknown cause in otherwise healthy women, it is important to consider the possibility of an ascending infection from the gynecological region.

By documenting this case, we underscore the significance of timely and accurate management of primary peritonitis caused by GAS. This emphasizes the importance of appropriate antibiotic treatment and avoiding unnecessary surgical intervention, which can significantly impact patient outcomes. Additionally, raising awareness of the potential association between hydrosalpinx and primary peritonitis can aid in early diagnosis and appropriate therapeutic decisions in similar clinical scenarios. Further research is warranted to enhance our understanding of this infrequent etiology and optimize patient care.
